# *Marigold Supercritical Extract* as Potential Co-adjuvant in Pancreatic Cancer: The Energetic Catastrophe Induced via BMP8B Ends Up With Autophagy-Induced Cell Death

**DOI:** 10.3389/fbioe.2019.00455

**Published:** 2020-01-24

**Authors:** Marta Gómez de Cedrón, Lamia Mouhid, Elena García-Carrascosa, Tiziana Fornari, Guillermo Reglero, Ana Ramírez de Molina

**Affiliations:** ^1^Molecular Oncology and Nutritional Genomics of Cancer, IMDEA-Food Institute, CEI UAM + CSIC, Madrid, Spain; ^2^Production and Characterization of Novel Foods Department, Institute of Food Science Research CIAL, CEI UAM + CSIC, Madrid, Spain; ^3^Production and Development of Foods for Health, IMDEA-Food Institute, CEI UAM + CSIC, Madrid, Spain

**Keywords:** precision nutrition, supercritical extract, marigold, cell bioenergetics, cancer

## Abstract

The recent development of powerful “omics” technologies (genomics, transcriptomics, proteomics, metabolomics, and lipidomics) has opened new avenues in nutritional sciences toward precision nutrition, which is a genotype-directed nutrition that takes into account the differential responses to nutritional interventions based on gene variation (nutrigenetics) and the effect of nutrients on gene expression (nutrigenomics). Current evidence demonstrates that up to one third of the deaths caused by cancer could be prevented by acting on key risk factors, with diet being one of the most important risk factors due to its association with obesity. Additional factors such as composition of gut microbiome, the immune system, and the nutritional status will have an impact on the final outcome. Nutrient components and bioactive compounds from natural sources can have an impact on cancer progression or even the risk of cancer development by regulating gene expression and/or associated risk factors such as obesity and chronic inflammation. Nowadays, among the different methods to produce natural extracts, the green technology of supercritical fluid extraction (SFE) is quite popular, with a special interest on the use of supercritical CO_2_ for the extraction of compounds with low polarity. The success of nutritional interventions based on the use of nutraceuticals requires several steps: (i) *in vitro* and preclinical demonstration of their antitumoral effects; (ii) knowledge of their mechanism of action and molecular targets, which will allow for identification of the specific subgroups of patients who will benefit from them; (iii) the study of genetic variants associated with the differential responses; and (iv) innovative approaches of formulations to improve the *in vivo* bioavailability of the bioactive ingredients. Herein, we investigate the antitumoral properties and mechanism of action of a supercritical CO_2_ extract from *Calendula officinalis*, commonly known as marigold (marigold SFE) in the context of pancreatic cancer. Mechanistically, marigold SFE induces the expression of BMP8B, which leads to an energetic catastrophe ending up with autophagy-induced cell death (AICD). As metabolic reprogramming is a well-recognized hallmark of cancer, the direct impact of marigold SFE on pancreatic cancer cell metabolism encourages further research of its potential as a coadjuvant in pancreatic cancer therapy. Finally, we discuss innovative formulation approaches to augment the clinical therapeutic potential of marigold SFE in nutritional interventions.

## Introduction

In the last years, there is great concern about the increase of chronic diseases related to metabolism including metabolic syndrome, cardiovascular disease, insulin resistance, obesity, and cancer. Importantly, it has been estimated that up to one third of cancer deaths could be prevented by modifying key risk factors, such as diet and exercise, due to their association with obesity (Parkin et al., 2011; Brown et al., 2018). Numerous epidemiological studies have shown that obesity increases the risk of developing different types of cancer (Lauby-Secretan et al., 2016), and accumulating evidence demonstrates that the overall metabolic state of an individual may contribute to the molecular alterations during the carcinogenic process. A key event during tumorigenesis is the reprogramming of the cancer energetic metabolism (Hanahan and Weinberg, 2011), and obesity-associated alterations (hormones, growth factors, cytokines, and other inflammatory molecules) may promote protumoral signals in pre- or neoplastic cells by interacting through their receptors and/or downstream intracellular signaling pathways (Gunter et al., 2015; Renehan et al., 2015; Murphy et al., 2018).

The recent development of powerful “omics” technologies [genomics, transcriptomics (Chen et al., 2007), proteomics (Sun et al., 2018), methylomics (Gaunt et al., 2016; Richmond et al., 2016), metabolomics (Shin et al., 2014; Würtz et al., 2014), lipidomics, microbiomics (Wang et al., 2018)] has opened new avenues in nutritional sciences toward *precision nutrition*. In the context of cancer, together with chemotherapy and/or radiotherapy used in the clinics, precision nutrition can help by means of the use of natural extracts, bioactive compounds, and nutritional recommendations to modulate gene expression and/or cancer-associated risk factors such as obesity, which will have an impact on the risk of developing this disease or its progression.

The success of such nutritional interventions requires several steps: (i) *in vitro* and preclinical demonstration of the antitumoral effects of selected extracts and/or bioactive compounds; (ii) the knowledge of their mechanism of action and molecular targets, which will identify the specific subgroups of patients who will benefit from them; (iii) the study of genetic variants associated with the differential responses to the intervention; and (iv) innovative approaches of new formulations to improve the *in vivo* bioavailability of the bioactive ingredients. Additional factors such as the gut microbiome composition, the immune system, and the nutritional status will refine the final outcome.

The use of phytochemicals and dietary-derived compounds in cancer prevention and/or treatment is well-demonstrated (Mouhid et al., 2017; Pan et al., 2017; Kumar et al., 2018; Imran et al., 2019; Tarasiuk and Fichna, 2019), such as taxol and camptothecin, which are extensively used in the clinics (Denda et al., 2019; Sanoff et al., 2019; Ulusakarya et al., 2019). Metabolic reprogramming in cancer not only supports the proliferation but also promotes malignancy and dissemination of cancer cells. In this regard, the exacerbated glucose uptake (Warburg effect) of proliferating cancer cells (Hanahan and Weinberg, 2011; Derle et al., 2018), the increased glutaminolysis supporting the proliferation and redox homeostasis (Li and Le, 2018; Bott et al., 2019), or lipid metabolism alterations associated with cancer dissemination (Currie et al., 2013; Luo et al., 2018; Munir et al., 2019) are well-documented.

One of the most popular sources of bioactive compounds are vegetables and plants. Phytochemicals exert important biological activities, such as anti-inflammatory, antihypertensive, antioxidant, anticarcinogenic, antidiabetic, or antiobesity.

For these reasons, current efforts are done toward the development of innovative methodologies to obtain bioactive compounds and/or natural extracts. Within the most promising methods, there is the green technology of supercritical fluids, with special use of supercritical CO_2_ in the extraction of compounds with low polarity. This technology can be assisted by distinct co-solvents in order to augment the performance of the extraction.

Herein, we have investigated the antitumoral properties and mechanism of action of a supercritical CO_2_ extract from *Calendula officinalis*, commonly known as marigold, in the context of pancreatic cancer.

Pancreatic cancer leads to the second position of deaths related to cancer worldwide. This cancer has poor prognosis, and the overall 5-year survival rate is <5%. Several risk factors in pancreatic cancer are obesity and chronic pancreatitis, tobacco smoking, alcohol intake, or diets with high intake of red meat (Ilic and Ilic, 2016; Gordon-Dseagu et al., 2017; Michaud, 2017;). Pancreatic cancer is often diagnosed at metastatic late stages due to the absence of indicators of illness. Although surgery remains the main beneficial treatment followed by chemotherapy (gemcitabine, 5-fluorouracil, irinotecan, and/or oxaliplatin) and radiation, as indicated previous, the patient's survival is limited.

Therefore, there is an urgent necessity to investigate on effective therapeutic strategies to improve patients' survival.

Recently, we have described the antitumoral properties of marigold supercritical fluid extract (marigold SFE) (Martin et al., 2016; García-Risco et al., 2017), in pancreatic cancer cell lines (Mouhid et al., 2018). Marigold SFE diminished the cell viability of pancreatic cancer cells in a dose-dependent manner, induced apoptotic cell death, increased the percentage of necrotic cells, inhibited the anchorage-independent cell growth, and synergized with the chemotherapeutic drug 5-fluorouracil (5-Fu), used in clinics.

Herein, we investigate the impact of marigold SFE on pancreatic cancer metabolism. By means of the use of the latest technology in the field of the analysis of cell bioenergetics, we demonstrate that marigold SFE targets the two main sources for energy production, mitochondrial oxidative respiration, and aerobic glycolysis. Marigold SFE leads to an energetic catastrophe, which ends up with autophagy-induced cell death (AICD). By means of gene expression microarray analysis, we identify the bone morphogenetic protein-8B (*BMP8B*) as a validated molecular target of marigold SFE.

The direct impact of marigold SFE on pancreatic cancer cell metabolism encourages further research of its potential as a complementary adjuvant in pancreatic cancer therapy. Finally, we discuss innovative formulation approaches to augment the clinical therapeutic potential of marigold SFE in nutritional interventions.

**Figure 1:**
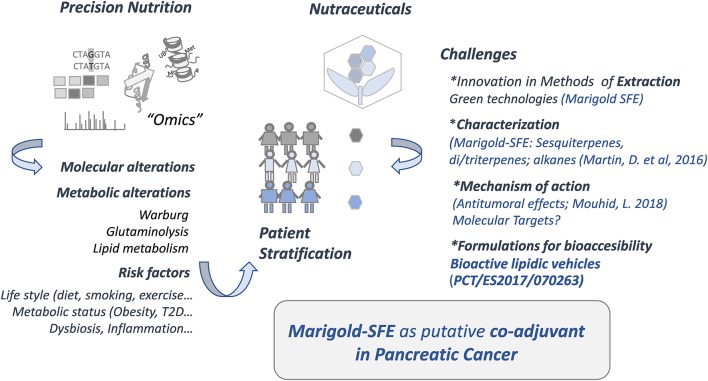
Precision nutrition and nutraceuticals.

Figure 1 summarizes the study workflow and challenges in the field of precision nutrition and nutraceuticals to be applied in cancer and other chronic diseases.

## Materials and Methods

### Marigold Supercritical Extract

Marigold SFE was obtained by CO_2_ supercritical fluid extraction (Thar Technology, model SF2000) in previously optimized process conditions (Martin et al., 2016). Briefly, a CO_2_ flux of 70 g/min at 140 bar and 40°C for 180 min was applied to 400 g of dried and grounded marigold in a 2-L cylinder. The resulting extract was collected with absolute ethanol, and the dissolvent was removed by means of rotary evaporation at 30°C. The extract chemical composition has been described in a preceding article (García-Risco et al., 2017).

### Cell Culture

Pancreatic cancer cells, MiaPaCa-2 and Panc-1, were obtained from American Type Culture Collection (Manassas, VA) and were cultured in Dulbecco's modified Eagle's medium (DMEM) supplemented with 10% fetal bovine serum (FBS; LONZA Iberica, S.A.) in an incubator with 95% humidity and 5% CO_2_.

### Extracellular Flux Analysis of the Acidification Rate and the Oxygen Consumption Rate

Mitochondrial oxidative respiration (Cell MitoStress Test) and aerobic glycolysis (Cell GlycoStress Test) were monitored with the XFe96 Cell Bionalyzer (Seahorse Biosciences, XFe96). Optimal cell density and drugs tritation were previously determined.

The dependency of the cells for aerobic glycolysis and oxidative phosphorylation was monitored after the injection of several modulators of both bioenergetic pathways.

Prior to the experiments, cells were pretreated with different doses of marigold SFE for 48 h. Non-treated cells were kept as controls.

For GlycoStress assay, 20,000 cells were plated in an XFe-96 well-plate and kept for 6 h in DMEM 10% FBS to allow the cells to attach. Then, the culture medium was changed to 0.2 mM glutamine XFe DMEM (5 mM Hepes) to starve the cells for 1 h. First basal extracellular acidification rate (ECAR) was measured (1–3 measurements). Glucose (10 mM) was injected (4–6 measurements) to the cells to determine glycolysis (this is the increased ECAR from basal starved situation after glucose addition). This indicates the capacity of the cells to use glucose. Next, maximal glycolytic capacity was monitored (7–9 measurements) after the injection of oligomycin, which inhibits the ATP production from the oxidative mitochondrial respiration. Finally, a third injection with 50 mM DG was done to specifically shut down aerobic glycolysis (10 to 12 measurements).

For MitoStress assay, 40,000 cells were plated in an XFe-96 well-plate, and cells were kept for 6 h in DMEM 10% FBS to allow the cells to attach. Then, the medium was changed to 10 mM glucose, 2 mM glutamine, and 1 mM pyruvate XFe DMEM (5 mM Hepes), and cells were incubated for 1 h at 37°C without CO_2_. Three different modulators of the mitochondrial respiration were sequentially injected. After basal oxygen consumption rate (OCR) determination (1–3 measurements), oligomycin (1 μM), which inhibits ATPase, was injected to determine the amount of oxygen dedicated to ATP production by mitochondria (3–6 measurements). To determine the maximal respiration rate or spare respiratory capacity, FCCP (carbonyl cyanide-4-(trifluoromethoxy)phenylhydrazone) was injected (0.4 μM) to free the gradient of H^+^ from the mitochondrial intermembrane space (7–9 measurements) and thus to activate maximal respiration. Finally, antimycin A and rotenone (0.5 μM) were added to completely inhibit the mitochondrial respiration (10–12 measurements).

### Measurement of Cellular ATP Content

For the quantification of the ATP content, the ATP-based assay CellTiter-Glo, Luminescent Cell Viability kit was used (Promega, Madison, WI, USA; Cat. #G7571) following the manufacturer's recommendations. Briefly, MiaPaca-2 cells were first pretreated (48 h) with marigold SFE at ½ × IC_50_, 1 × IC_50_, and 2 × IC_50_, with IC_50_ being equal to 39.8 (±_4.6) μg/ml as previously described (Mouhid et al., 2018). Non-treated cells were kept as controls. A total of 10,000 MiaPaca-2 cells were plated in 96-well-clear bottom black polystyrene plates.

### Western Blot

After 48 h of treatment with marigold SFE, MiaPaca-2 or Panc-1 pancreatic cancer cells were washed and detached using trypsin. The RIPA buffer with protease and phosphatase inhibitors was used to lyse the cells. After centrifugation (12,000 g) for 10 min at 4°C, supernatants were recovered. Proteins were denatured and loaded into a 4–15% Mini-Protean TGX Precast Protein Gel (BioRad) for electrophoretic separation, and transferred onto a nitrocellulose membrane. Membranes were blocked using 5% no-fat dry milk in TBS 0.05% Tween-20. Primary antibodies were incubated overnight at 4°C, and secondary antibodies were incubated for 1 h. β-Actin or Ponceau staining was used as the loading control. The primary antibodies used were rabbit polyclonal anti-LC3 (PM036, 1:1,000 dilution, MBL), rabbit monoclonal anti-phospho-AMPKα (T172) (40H9, 1:1,000 dilution, Cell Signaling), and rabbit monoclonal anti-AMPKα antibody (23A3, 1:1,000 dilution, Cell Signaling). For loading controls, β-actin (Sigma-Aldrich) or Ponceau staining was used. Signals were visualized using ECL plus (GE Healthcare, Little Chalfont, UK).

### Immunofluorescence

MiaPaca-2 cells were spread in M24 well-plates on top of glass coverslips for o/n. Then cells were treated with different doses of marigold SFE (1 × IC_50_, 2 × IC_50_) for 48 h. Non-treated cells were kept as the control. Cells were fixed in 4% PFA/PBS for 10 min at RT. Then cells were permeabilized with 100 μg/ml of digitonin for 20 min at RT. After washing with PBS, anti-LC3 antibody (1:1,000) was added and incubated for 1 h at RT. After washing, 1:500 Alexa Fluor 488 Goat Anti-rabbit IgG (Invitrogen, A11008) was incubated for 30 min at RT. DAPI was incubated for 5 min at RT. Positive controls were incubated for 6 h in Hank's solution at 37°C. E64d and pepstatin A treatments were done at 10 μM and 10 μg/ml, respectively.

### Quantitative Real-Time Polymerase Chain Reaction

MiaPaca-2 and Panc-1 cells (0.35 × 10^6^ cells) were treated with marigold SFE for 48 h at different doses: ½ IC_50_, 1 × IC_50_, 2 × IC_50_, with IC_50_ values being equal to 39.8 μg/ml (±_4.6) and 43.2 μg/ml (±7.9), respectively, for MiaPaca-2 and Panc-1, as previously described (Mouhid et al., 2018). Non-treated cells were kept as controls.

Total RNA was extracted with Tri Reagent (Sigma). One microgram of RNA was reverse-transcribed with the High Capacity RNA-to-cDNA Master Mix system (Life Technologies). Quantitative polymerase chain reaction (qPCR) was performed in the 7900HT Real-Time PCR System (Life Technologies) using the VeriQuest SYBR Green qPCR Master Mix (Affymetrix, Santa Clara, CA, USA), and Taqman probes were used: Hs01629120_s1, Hs01029413_m1, Hs00245183_m1, and Hs99999901_s1 for *BMP8B, TFAP2A, ZFP36L1*, and 18S, respectively; or oligos in the case of the epithelial-to-mesenchymal transition (EMT), stemness and endoplasmic reticulum (ER) stress markers (Table S1 displays the list and sequences of the primers used). The 2^−ΔΔCt^ method was applied to calculate the relative gene expression (Livak and Schmittgen, 2001).

### Microarray Gene Expression Assay

MiaPaca-2 cells were plated (2 × 10^6^) in p100 plates and 12 h later were treated for 48 h with 30 and 70 μg/ml of marigold SFE. Non-treated cells were kept as controls. Total RNA was isolated with the RNeasy Mini Kit (Qiagen Iberica). The microarray gene expression analysis between control and treated cells was performed by the Genomic Service of the National Center of Biotechnology (CNB-Madrid, Spain). After RNA integrity validation, RNAs were reverse transcribed and fluorescently tagged with the one-color Low Input Quick Amp Labeling Kit (Agilent Technologies). The microarray gene expression platform used was the Agilent Sure Print G3 Human 8 × 60 K (Whole Human Genome Microarray Kit).

### *BMP8B* Depletion With si-RNA-Pools

Cells (0.25 × 10^6^) were plated in six-well-plates with si-RNA pools (siTOOLs Biotech GmbH, Planegg, Germany) against the human *BMP8B* mRNA to transiently deplete the expression of *BMP8B*. Lipofectamine RNAimax was used (Life Technologies, Darmstadt, Germany) to transfect MiaPaca-2 and Panc-1 pancreatic cancer cells for 4 to 6 h. After that, cells were treated with the indicated doses of marigold SFE for 48 h. Cells transfected with a negative control siPOOL against sequences not found in human were kept as controls. The functional role of *BMP8B* depletion on cell bioenergetics was further analyzed.

### Invasion Assays

Matrigel-coated chambers (BD Biosciences Madrid, Spain) were used for invasion assays. Images were obtained with the Olympus CKX41 microscope. The analysis was done with the GETIT software.

### Statistical Analysis

Microarray gene expression data were analyzed with FIESTA software (version 1.0) Statistical analyses were performed using Limma (Smyth, 2005). A *P* < 0.05 was set to establish the limit of significance and a change of two-fold or higher fold to consider upregulation or repression of the genes. One-way analysis of variance (ANOVA; Bonferroni *post-hoc* test) was used to determine qPCR differences between gene expression and invasion through Matrigel-coated chambers. *P < 0.05, **P < 0.01, and ***P < 0.005 indicate significant differences. GraphPad Prim 8.0.1 statistical software was used for all statistical analyses.

## Results

### Marigold SFE Diminishes the Cell Bioenergetics of Pancreatic Cancer Cells

In the last years, there is an increased interest on targeting the altered cancer metabolism. In a previous work, we have described the antitumoral effects of marigold SFE in pancreatic cancer cell lines (marigold SFE inhibited cell viability, induced apoptosis, and augmented the percentage of necrotic cells) (Mouhid et al., 2018). For this reason, herein, we aimed to investigate if marigold SFE could target cancer cell metabolism, and more specifically the energetic metabolism (glycolysis and mitochondrial respiration) of pancreatic cancer cells.

### Marigold SFE Diminishes the Mitochondrial Respiration of Pancreatic Cancer Cells

First, we investigated the impact of marigold SFE on the mitochondrial oxidative respiration of pancreatic cancer cells. By means of the Extracellular Flux Bioanalyzer (Seahorse Bioscience), we monitored the OCRs after the sequential addition of different drugs, which regulate the mitochondrial function.

As we have previously described (Mouhid et al., 2018), the IC_50_ (dose concentration that inhibits 50% of cell proliferation) of marigold SFE after 48 h of treatment was 39.8 (±_4.6) μg/ml for MiaPaca-2 cells. The LC_50_ (μg/ml) (concentration needed for 50% cell death) after 48 h of treatment was 78.5 (± 1.4) μg/ml. For this reason, the study of cell bioenergetics (mitochondrial oxidative phosphorylation and aerobic glycolysis) was performed after treatment of MiaPaca-2 cells for 48 h with different doses of marigold SFE, corresponding to ½ × IC_50_ (¼ × LC_50_), 1 × IC_50_ (½ × LC_50_), and 2 × IC_50_ (1 × LC_50_). Non-treated cells were kept as controls. Importantly, before running the experiments, the very same number of non-treated cells and pretreated cells (40,000 cells per well) was plated in an XFe Seahorse plate in complete media (DMEM, 10% FBS, for 4 h to allow the cells to attach), without any treatment, in order to compare the cell bioenergetics only of viable cells. Then, the medium of the cells was changed to the non-buffered XFe base media supplemented with 10 mM glucose, 2 mM glutamine, and 1 mM pyruvate, and cells were incubated for 1 h at 37°C without CO_2_.

**Figure 2:**
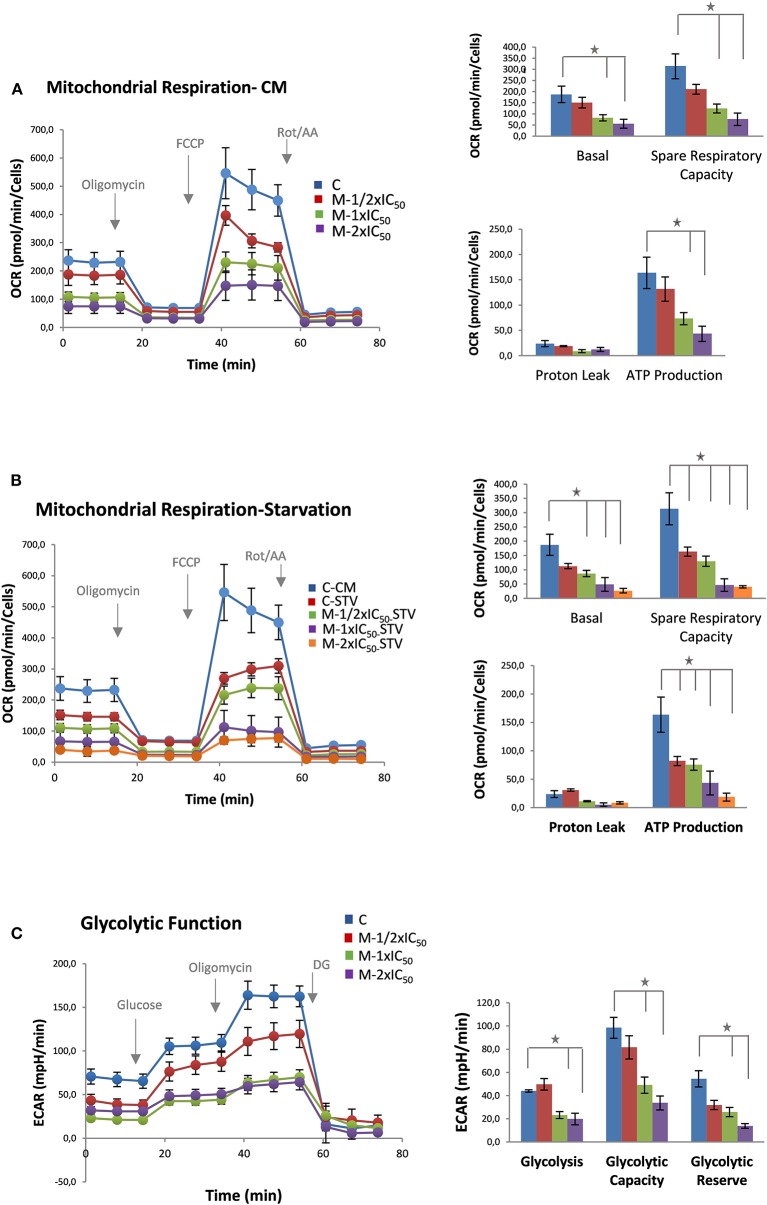
Marigold supercritical fluid extraction (SFE) diminishes cell bioenergetics. **(A)** Mitochondrial respiration analysis by flux analysis of the oxygen consumption rate (OCR) of MiaPaca-2 cells vs. marigold SFE pretreated cells (½ × IC_50_, 1 × IC_50_, 2 × IC_50_) in complete media (10 mM glucose, 2 mM glutamine, 1 mM pyruvate). Basal respiration rate, spare respiratory capacity, ATP production, and proton leak are shown. **(B)** Mitochondrial respiration analysis by flux analysis of the OCR in MiaPaca-2 non-treated cells vs. marigold SFE pretreated cells for 48 h prior to the experiment, when kept in low glucose (2.5 mM glucose, 2 mM glutamine, 1 mM pyruvate). A control of non-treated cells in complete media is shown for comparison with control non-treated cells in conditions of low glucose. **(C)** Glycolytic activity of MiaPaca-2 pancreatic cancer cells. Comparison of the glycolytic function of MiaPaca-2 cells vs. marigold SFE pretreated cells (½ × IC_50_, 1 × IC_50_, 2 × IC_50_) for 48 h prior to the experiment, in conditions of absence of glucose for 1 h (2 mM glutamine). Glycolysis [difference from extracellular acidification rate (ECAR) value in the presence of glucose and ECAR value in starved cells], glycolytic reserve (difference between ECAR after ATPase inhibition and ECAR after glucose injection), and glycolytic capacity (sum of glycolysis and glycolytic reserve) are shown. Representative assays of three experiments. Each experiment contains six replicates per treatment. *P < 0.05.

As it can be observed in Figure 2A (left panel, bioenergetic profile), MiaPaca-2 pancreatic cancer cells pretreated with marigold SFE showed a reduced basal respiration rate compared with control non-treated cells (measurements 1–3) at all the doses tested. After injection of oligomycin, in order to estimate the OCR dedicated to ATP production, marigold SFE pretreated cells displayed reduced levels of ATP compared to control non-treated cells (measurements 4–6). The maximal respiration rate (measurements 7–9), after the injection of FCCP, was also affected in marigold SFE pretreated cells. Finally, rotenone and antimycin A, inhibitors of complexes I and III of the electron transport chain, respectively, were injected to shut down the OCR due to mitochondrial oxidative phosphorylation (measurements 10–12). These results indicate that marigold SFE clearly compromises mitochondrial respiration.

More dramatic results were obtained when the same experiment was performed in a reduced glucose condition (2.5mM glucose) (Figure 2B).

These results indicate that, even at the lowest dose (20 μg/ml) of treatment, where cell viability is not highly compromised (¼ of the LC_50_ dose), marigold SFE compromises the cell bioenergetics of MiaPaca-2 pancreatic cancer cells.

### Marigold Diminishes Aerobic Glycolysis of Pancreatic Cancer Cells

To analyze the effect of marigold SFE on aerobic glycolysis, we monitored the ECAR, an indirect readout of the L-lactate production, after sequential injection of modulators of the aerobic glycolysis. MiaPaca-2 cells were pretreated for 48 h with three different doses of marigold SFE corresponding to ½ × IC_50_, 1 × IC_50_, and 2 × IC_50_, with IC_50_ being equal to 39.8 (± 4.6) μg/ml. Non-treated cells were kept as controls. Then, 20,000 cells were plated per condition (six replicates for control and six replicates for the different doses of marigold SFE pretreated cells) in complete media (DMEM, 10% FBS), without treatments, for 4 h to allow the cells to attach. Then, the culture medium was changed to XFe base media supplemented with 2 mM glutamine without glucose and pyruvate in non-buffered media adjusted to pH 7.4. Cells were kept for 1 h at 37°C in an incubator without CO_2_.

As it can be observed in Figure 2C (left panel), basal ECAR of marigold SFE pretreated cells (1 to 3 measurements) was reduced compared to control non-treated cells. Next, we injected glucose (10 mM final concentration) to monitor the cells' ability to upregulate glycolysis when glucose is available. After the glucose injection, marigold SFE pretreated cells had diminished levels of ECAR compared to control cells (3–6 measurements), indicating a reduced capacity for glycolysis Then, oligomycin was injected to block the ATP production from the mitochondria and so to determine the maximal glycolytic capacity. Marigold SFE pretreated cells presented reduced maximal ECAR levels compared to the control cells (measurements 6–9).

Finally, 2-DG was injected (50 mM final concentration) to shut down aerobic glycolysis and to determine the non-glycolytic ECAR.

These results indicate that marigold SFE treatment also compromises aerobic glycolysis.

Different from other systems where there is bioenergetic plasticity between mitochondrial oxidative respiration and aerobic glycolysis, herein, marigold SFE diminished both bioenergetic pathways.

### Marigold SFE Leads to AMPK Activation and Augments the Autophagy Marker LC3-II

**Figure 3:**
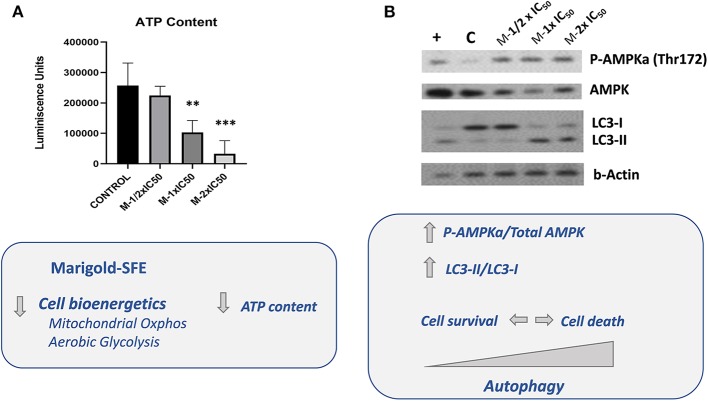
Marigold SFE leads to AMPK activation and increased markers of autophagy (LC3-II). **(A)** Measurement of the intracellular ATP content. Luminescence units were registered for marigold SFE pretreated cells (½ × IC_50_, 1 × IC_50_, and 2 × IC_50_) and compared to control non-treated cells. **(B)** Western blot analysis of P-(Thr172) AMPK and Total AMPK protein levels, and LC3 protein. Cells incubated for 6 h in low glucose (2.5 mM) were kept as a positive control of AMPK activation and LC3 lipidated form (LC3-II). **P < 0.01, ***P < 0.005.

As the two major bioenergetic pathways, mitochondrial oxidative phosphorylation (Figures 2A,B) and aerobic glycolysis (Figure 2C), were compromised in marigold SFE-treated cells compared to control non-treated cells, we wanted to delve deep into the energetic depletion as a plausible major cause of pancreatic cancer cell death. Cell death can occur by different mechanisms, including apoptosis, necrosis, or autophagy. Nevertheless, ATP depletion is involved in all processes that mediate cell death. Thus, we quantified the intracellular ATP content after the treatment with marigold SFE. MiaPaca-2 cells were pretreated for 48 h with marigold SFE at ½ × IC_50_, 1 × IC_50_, and 2 × IC_50_. Non-treated cells were kept as controls. A total of 10,000 cells of each condition were replated, in complete media without any treatment, for 6 h before the ATP content quantification. As shown in Figure 3A, the cellular ATP content was reduced in marigold SFE pretreated cells in a dose-dependent manner, being statistically significant at doses of 1 × IC_50_ and 2 × IC_50_.

The active phosphorylated form of AMPK (P-AMPKα) at residue Thr172 in the catalytic loop is required to inhibit essentially all anabolic pathways that promote cell growth. As it can be observed in Figure 3B, marigold SFE pretreated cells displayed increased levels of the phosphorylated active form of AMPK [P-AMPKα (Thr172)].

In response to various cellular stresses, including glucose starvation and/or ER stress, AMPK can induce autophagy. To evaluate induction of autophagy, by means of Western blot, we analyzed the conversion of the soluble form of LC3 (LC3-I) to the lipidated and autophagosome-associated form (LC3-II), which is considered one of the hallmarks of autophagy (Rubinsztein et al., 2007; Klionsky et al., 2016). As it is shown in Figure 3B, marigold SFE pretreated cells displayed increased LC3-II levels compared to control non-treated cells.

Similar results were obtained in Panc-1 pancreatic cancer cells after treatment with marigold SFE (Figure S1).

### Marigold SFE Induces a Dynamic Autophagy Flux

AMPK activation not only regulates the initiation of autophagy when the intracellular ATP levels are decreased, but also is required for the autophagosome maturation and lysosomal fusion (Jang et al., 2018).

**Figure 4:**
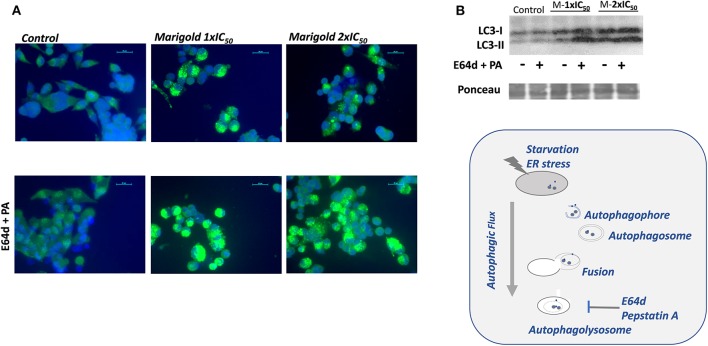
Effect of marigold SFE on induction of autophagy. **(A)** LC3 immunostaining. Representative images of control and marigold SFE-treated cells (48 h) at two different doses corresponding to 1 × IC_50_ and 2 × IC_50_, in the absence (left panel) or presence of E64d (10 μM) and pepstatin A (PA; 10 μg/ml) (right panel). Scale bar: 20 μm. **(B)** Effect of marigold SFE on LC3 lipidation in MiaPaca2 cells pretreated at two different doses corresponding to 1 × IC_50_ and 2 × IC_50_ for 48 h and control non-treated cells, in the presence/absence of E64d (10 μM) and PA (10 μg/ml).

By means of immunofluorescence, the occurrence of LC3-positive dots was observed after marigold SFE treatments compared to non-treated cells. Importantly, the addition of the lysosomal protease inhibitors, E64d and pepstatin A, which block the last steps of autophagy (Klionsky et al., 2016), augmented the marigold SFE-induced accumulation of LC3 dots and intensity, confirming the dynamic autophagy flux induced by marigold SFE (Figure 4A). Moreover, these results were confirmed by Western blot, where marigold SFE in the presence of E64d and pepstatin A displayed increased levels of the lipidated LC3-II marker (Figure 4B).

### Marigold SFE Augments Markers of ER Stress and AICD

Since autophagy has been implicated in cell survival or cell death, depending on the intensity, and duration, we wanted to investigate if the increased autophagic flux could be implicated in the pancreatic cancer cell death induced by marigold SFE.

A role of ER stress has been described, when energy is depleted, in the activation of the autophagic flux. Depending on the duration and intensity, it may end up with AICD. We wanted to evaluate this possibility by analyzing the effect of marigold SFE in the expression levels of *BIP*, which is a marker of ER stress, and *CHOP*, which is a marker of AICD.

**Figure 5:**
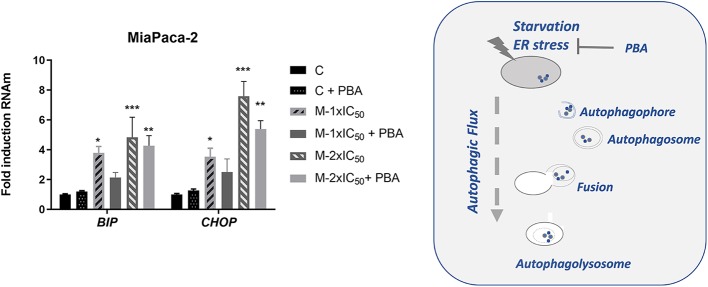
Marigold SFE induces the expression of *BIP* and *CHOP*. Effect of marigold SFE on the expression levels of *BIP* and *CHOP* of MiaPaca-2 cells pretreated at two different doses corresponding to 1 × IC_50_ and 2 × IC_50_ for 48 h, in the presence or absence of PBA. Non-treated cells were kept as controls. Data represent the mean ±_S.E.M of three independent experiments, each one performed in triplicate. Asterisks indicate statistical differences in treated cells in comparison with control (DMSO); *P < 0.05, **P < 0.01, ***P < 0.005.

As shown in Figure 5, marigold SFE increased the expression levels of *BIP* and *CHOP* in a dose-dependent manner. Importantly, treatment with PBA, which is known to alleviate the ER stress, also diminished the expression levels of *CHOP*.

### *BMP8B* Is a Molecular Target of Marigold SFE

To identify putative molecular targets of marigold SFE, a comparative gene expression microarray (G2519F-026652 Human Gene Expression v2 4x44K Microarray) was performed (Link to raw data: https://www.ncbi.nlm.nih.gov/geo/query/acc.cgi?acc=GSE124043).

**Figure 6:**
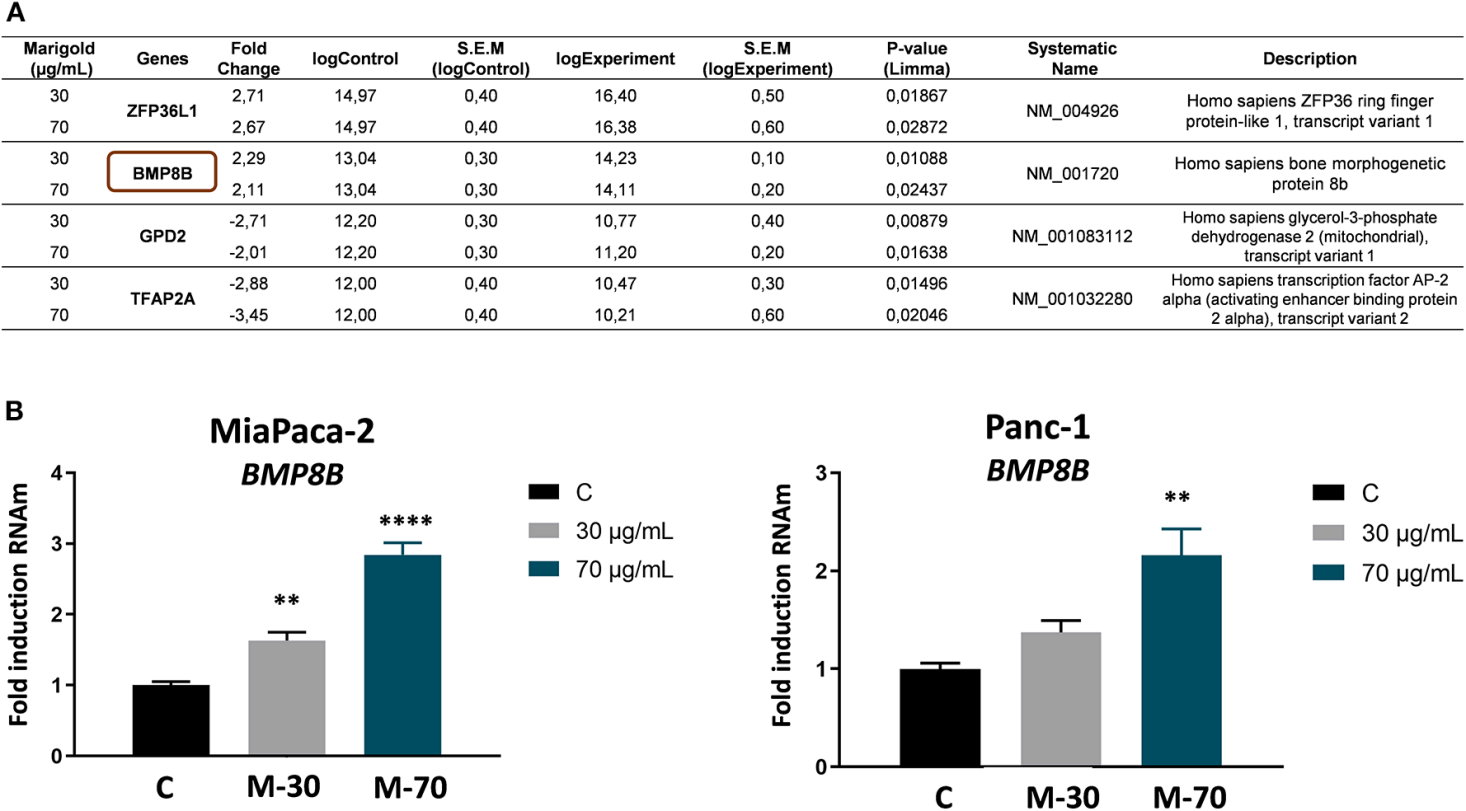
*BMP8B* is a molecular target of marigold SFE. **(A)** Microarray data of differentially expressed genes after treatment of MiaPaCa-2 pancreatic cancer cell line with 30 and 70 μg/ml marigold SFE for 48 h. Data represent the value of the most significant probe for three independent experiments for each condition. Genes with a statistically significant difference (*P* value < 0.05) and more than two-fold absolute change variation compared to control (DMSO) are shown. **(B)** Validation of the upregulation of *BMP8B* after marigold SFE treatment in two pancreatic cancer cell lines, MiaPaca-2 and Panc-1. Non-treated cells were kept as controls. Data represent the mean ± S.E.M. of three independent experiments, each one performed in triplicate. Asterisks indicate statistical differences in treated cells in comparison with control (DMSO). **P < 0.05, ****P < 0.001 indicate statistically significant differences refered to the control C.

For this, MiaPaca-2 cells were treated with two doses of marigold SFE (30 and 70 μg/ml) for 48 h, to be compared to control non-treated cells. Genes whose expression was significantly changed at the two doses (*P* < 0.05), with a >two-fold change compared to control cells, are listed in Figure 6A. By means of quantitative real-time PCR (RT-qPCR) analysis, the upregulation of *BMP8B* after marigold SFE treatment in a dose-dependent manner was validated (30 and 70 μg/ml) (Figure 6B, left panel). We obtained similar results with another pancreatic cancer cell line, Panc-1, which has been described to be more aggressive compared to MiaPaCa-2 cells (Yang et al., 2011) (Figure 6B, right panel).

Upregulation of *BMP8B* has been demonstrated to diminish cell invasion and tumor growth in pancreatic xenografts (Cheng et al., 2014). In line with this, marigold SFE inhibited cell invasion through Matrigel-coated chambers, as well as EMT and stemness markers in a dose-dependent manner (Figure S2).

### Silencing *BMP8B* Counteracts the Effect of Marigold SFE on Mitochondrial Oxidative Phosphorylation

Bone morphogenetic proteins (BMPs) have been described to have an impact on the systemic energy balance by targeting brown and white adipose tissues. BMP8B central administration induces thermogenesis and augments the core temperature, leading to weight loss (Whittle et al., 2012). To demonstrate the functional link of BMP8B after marigold treatment with the observed effects on cell bioenergetics and autophagy, we performed rescue experiments to diminish *BMP8B* RNAm levels. For this, MiaPaca-2 pancreatic cancer cells were transfected with si-RNA pools against the human *BMP8B* mRNA (siTOOLs Biotech GmbH). Twenty-four hours later, cells were treated with two different doses of marigold SFE for 48 h [d1 = 1 × IC_50_ and d2 = 2 × IC_50_, with IC_50_ being 39.8 (±4.6) μg/ml for MiaPaca2]. Cells transfected with a negative control siPOOL were kept as controls.

**Figure 7:**
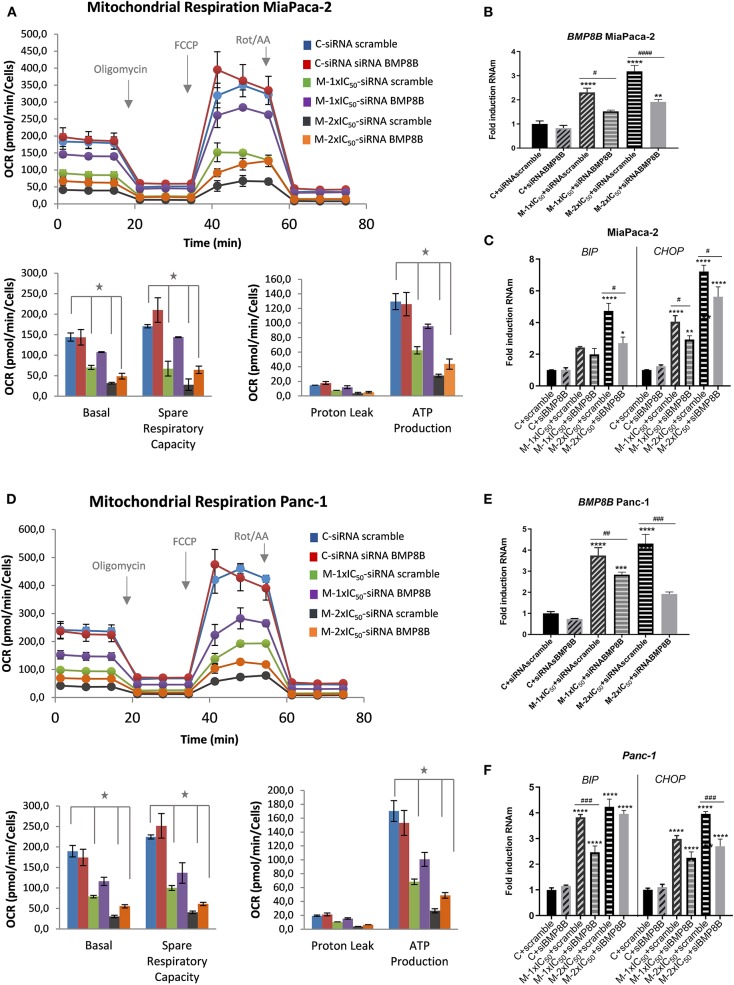
Depletion of *BMP8B* counteracts the effect of marigold SFE on the inhibition of mitochondrial oxidative phosphorylation. **(A)** Mitochondrial respiration analysis by flux analysis of the OCR in MiaPaca-2 transfected with si-RNA pools against *BMP8B* or si-RNA-scramble, in non-treated cells and marigold SFE-treated cells (1 × IC_50_, 2 × IC_50_). Lower panels: basal respiration rate, spare respiratory capacity, ATP production, and proton leak. **(B)** Quantitative real-time PCR (qRT-PCR) validation of the partial inhibition of *BMP8B* by means of si-RNA pools against BMP8B in MiaPaca-2 cells. **(C)** Effect of marigold SFE on the expression levels of *BIP* and *CHOP* in MiaPaca-2 cells transfected with si-RNA pools against *BMP8B* or si-RNA-scramble. **(D)** Mitochondrial respiration analysis by flux analysis of the OCR in Panc-1 cells transfected with si-RNA pools against *BMP8B* or si-RNA-scramble, in non-treated cells and marigold SFE-treated cells (1 × IC_50_, 2 × IC_50_). **(E)** qRT-PCR validation of partial inhibition of BMP8B by means of si-RNA pools against *BMP8B* in Panc-1 cells. **(F)** Effect of marigold SFE on the expression levels of *BIP* and *CHOP* in Panc-1 cells transfected with si-RNA pools against *BMP8B* or si-RNA-scramble. *Indicates statistical differences referred to control non-treated cell transfected with siRNA scramble: *P < 0.01; **P < 0.05, ***P < 0.005, ****P < 0.001. ^#^Indicates statistical differences between siRNA BMP8B and siRNA scramble transfected cells for each condition: ^#^*P* < 0.05, ^##^*P* < 0.01, ^###^*P* < 0.005, ^####^*P* < 0.001.

Importantly, silencing *BMP8B* was able to partially counteract the effect of marigold SFE on the inhibition of the mitochondrial oxidative phosphorylation (Figure 7A). RT-qPCR confirmed the partial downregulation of *BMP8B* in MiaPaca-2 transfected cells with si-RNA pools against *BMP8B*, but not with the si-RNA scramble (Figure 7B). In addition, we also found that diminishing *BMP8B* also correlated with the levels of LC3-II, which were also diminished in siRNA *BMP8B* transfected cells compared to the si-RNA scramble transfected cells (Figure S3). These results suggest that the energetic depletion is linked to the activation of autophagy.

To determine if the activation of autophagy could be linked to the observed effects on cell viability, we analyzed the markers of ER stress and its downstream mediator of apoptotic cell death CHOP. As shown in Figure 7C, reducing *BMP8B* in marigold-treated cells was able to alleviate, to some extent, ER stress (*BIP* marker) and *CHOP* expression levels.

These results were also validated in Panc-1 pancreatic cancer cells (Figures 7D–F).

## Discussion

There is great concern about the increase in chronic diseases related to metabolism such as obesity, insulin resistance, cardiovascular diseases, and cancer. Moreover, current evidence demonstrates that up to one third of cancer deaths could be prevented by modifying key risk factors, with diet and exercise being among the most important due to their association with obesity (Parkin et al., 2011; Brown et al., 2018). Although the biology that connects metabolic dysfunction with cancer development at either cellular or systemic level is not fully understood, accumulating evidence suggests that the overall metabolic state of an individual may contribute to the molecular alterations during carcinogenesis.

In the context of precision nutrition, nutrient components, extracts, and bioactive compounds from natural sources (nutraceuticals) can have an impact on cancer initiation and/or on cancer progression by regulating gene expression and/or associated risk factors such as obesity and chronic inflammation.

The reprogramming of energy metabolism is a key event in tumorigenesis (Hanahan and Weinberg, 2011), and efforts are oriented to develop therapeutic strategies toward the altered cancer metabolism and/or associated molecular alterations. Dysregulated metabolism also has an impact on the oncogenic pathways of cancer, and diet is a contributing factor to supply cancer metabolic requirements.

Vegetables and plants are among the most popular sources to obtain bioactive compounds. Phytochemicals exert numerous biological activities, such as anti-inflammatory, antihypertensive, antioxidant, anticarcinogenic, antidiabetic, or antiobesity. Thus, targeted nutritional interventions may contribute to enhancing cancer therapy and survival of cancer patients.

The success of such nutritional interventions requires several steps: (i) *in vitro* and preclinical demonstration of the antitumoral effects of selected extracts and/or bioactive compounds; (ii) the knowledge of their mechanism of action and molecular targets, which will identify the specific subgroups of patients that will benefit from them; (iii) the study of genetic variants associated with the differential responses to the intervention; and (iv) innovative approaches of new formulations to improve the *in vivo* bioavailability. Moreover, additional factors such as the gut microbiome composition, the immune system, and the nutritional status will refine the final outcome.

Innovative methodologies are being developed to obtain new bioactive compounds or extracts, which potentially could target the altered cancer metabolism. Within the most promising methods, the green technology of SFE is quite popular nowadays, with special use in the extraction of compounds with low polarity, which are soluble in supercritical CO_2_. This technology can be assisted by distinct co-solvents such as ethanol, which may also enhance the yield of extraction.

The antitumoral activities of different extracts obtained from marigold have been previously evaluated. Thus, extracts obtained with polar solvents (i.e., hexane, chloroform, ethyl acetate, and methanol) have been shown to display cytotoxic activity against breast cancer cell lines (Abutaha et al., 2019), and extracts obtained by laser have also been evaluated in pancreatic cancer (Fryer et al., 2011), leukemia, and fibrosarcoma cell lines (Ukiya et al., 2006), as well as ethyl alcohol extracts in melanoma (Preethi et al., 2010). To our knowledge, the antitumoral activities of supercritical extracts obtained from marigold have not been previously described.

For this reason, herein, we have investigated the antitumoral properties and mechanism of action of a supercritical CO_2_ extract from *C. officinalis*, commonly known as marigold in the context of *pancreatic cancer*, where the 5-year overall survival rate is <5%.

We have previously described the antitumoral properties of marigold SFE (Martin et al., 2016; García-Risco et al., 2017) in pancreatic cancer cell lines (Mouhid et al., 2018). Herein, we aimed to investigate the impact of marigold SFE on pancreatic cancer metabolism. By means of the use of the latest technology in the field of cell bioenergetics, we analyzed the impact of marigold SFE on mitochondrial oxidative phosphorylation and aerobic glycolysis. We functionally demonstrate that marigold SFE targets cell bioenergetics (Figure 2) and diminishes the ATP content of pancreatic cancer cells (Figure 3) and increases the autophagy flux (Figure 4, Figure S1). We also show that marigold SFE increases the ER stress and markers of AICD. Importantly, alleviating the marigold SFE-induced ER stress with PBA diminishes the AICD (Figure 5).

Gene expression microarray analysis allowed for identifying *BMP8B* as a validated molecular target of marigold SFE (Figure 6). BMP8B has been proposed to act as a tumor suppressor in pancreatic cancer, leading to the inhibition of invasion and tumor growth of pancreatic cancer xenografts (Cheng et al., 2014). In accordance with this, marigold SFE inhibited cell invasion as well as markers of EMT and stemness (Figure S2).

BMP8B, which is expressed in mature brown adipocytes, has also been demonstrated to amplify the thermogenic response in brown adipose tissue influencing the systemic energy balance. Moreover, BMP8B-deficient mice displayed a reduced thermogenic response and an impairment in diet- and cold-induced thermogenesis (Whittle et al., 2012). On the contrary, BMP8B administration induced thermogenesis and increased the core temperature, as well as promoted weight loss. Interestingly, this effect results from the sympathetic activation of brown adipose tissue, without any change in the feeding behavior (Livak and Schmittgen, 2001).

Importantly, depleting BMP8B by means of siRNAs counteracts, at least partially, the effect of marigold SFE on the LC3-II marker (Figure S3), with improvements on cell bioenergetics (indirect readout of cell viability recovery) (Figures 7A,D) and also alleviating ER stress and CHOP apoptotic markers (Figures 7C,E). These findings suggest that marigold promotes autophagic cell death via ER stress in pancreatic cancer cells, although we cannot exclude that autophagy and apoptosis may occur simultaneously. A deeper study on autophagy downstream players requires further investigation.

Further studies for the identification of individual bioactive constituents in the SFE marigold extract, and their individual or associated roles in pancreatic cancer metabolism are still necessary before conducting preclinical trials.

In the field of nutraceuticals, additional challenges are directed to augment the bioavailability of the bioactive compounds during the gastrointestinal digestion. Despite their bioactive properties, the use of phytochemicals in the clinics is still limited, mainly due to their poor bioavailability, and new formulations are being develop (Mouhid et al., 2017). The use of bioactive lipids as delivery systems is a very interesting strategy not only to increase the bioavailability of phytochemicals but also to exert additional benefits due to their intrinsic bioactivity (Mouhid et al., 2017). In this regard, this delivery system has been gone under patent by the group, and a formulation based on the use of alkylglycerols as a lipidic vehicle has demonstrated to synergize with rosemary SFE to exert antitumoral activity in colon cancer both *in vitro* and *in vivo* (PCT/ES2017/070263). Furthermore, in a recent clinical trial, this formulation based on the combination of alkylglycerols and rosemary SFE has demonstrated immunomodulatory and molecular effects with potential benefits in colorectal cancer (Clinical Trial Registry number: NCT03492086 http://clinicaltrials.gov/), suggesting that a similar formulation could be applied to marigold in pancreatic cancer as the next step.

Additional challenges are oriented to understand how diet might have an impact on cancer development through the gut microbiota composition, the metabolites derived from them, and the immune system. The equilibrium between inflammation and tolerance in the gut can modulate precancerous lesions to progress or not into cancer (Arpaia et al., 2013). The nutritional status of individuals also has an impact by triggering inflammation and affecting the systemic energy balance.

## Future Directions

Herein, it is demonstrated that marigold SFE induces BMP8b in pancreatic cancer leading to AICD. These results open new stimulating scenarios for the potential applications of marigold SFE. BMP8b, which is secreted by brown and beige adipocytes, has been demonstrated to activate thermogenesis, to enhance energy dissipation, and to increase the sympathetic innervation and vascularization of AT (Pellegrinelli et al., 2018). Thus, induction of BMP8b by marigold SFE could be an interesting strategy for tackling obesity and for promoting metabolic health. To further confirm these potential applications, a formulation of marigold with bioactive lipids will be performed to undergo clinical validation.

## Data Availability Statement

The datasets generated for this study can be found in the https://www.ncbi.nlm.nih.gov/geo/query/acc.cgi?acc=GSE124043.

## Author Contributions

MG, LM, and EG-C performed the experiments. TF obtained and provided marigold SFE. MG wrote the paper. MG and AR designed the research plan and supervised the study. GR and AR supported the experiments and revised the manuscript. All authors read and approved the final version of the manuscript.

### Conflict of Interest

The authors declare that the research was conducted in the absence of any commercial or financial relationships that could be construed as a potential conflict of interest.
