# Supplementary material for: Marigold Supercritical Extract as Potential Co-adjuvant in Pancreatic Cancer: The Energetic Catastrophe Induced via BMP8B Ends Up With Autophagy-Induced Cell Death
Source: Front Bioeng Biotechnol. 2020 Jan 24;7:455. doi: 10.3389/fbioe.2019.00455 (PMC6992545; doi:10.3389/fbioe.2019.00455)
Supplement: Supplementary file 1 [file Table_1.DOCX]

**Table Supplementary 1**

List of primers

| **Name** | **Sequence** |  |  |
| --- | --- | --- | --- |
| *E-cadh- Fw* | 5'-GAA CGC ATT GCC ACA TAC AC-3' | | |
| *E-cadh- Rv* | 5'-GAA TTC GGG CTT GTT GTC AT-3' | | |
| *N-cadh- Fw* | 5'-CGG TTT CAT TTG AGG GCA CA-3' | | |
| *N-cadh- Rv* | 5'-TTG GAG CCT GAG ACA CGA TT-3' | | |
| *Vim- Fw* | 5'-GAG TCC ACT GAG TAC CGG AG-3' | | |
| *Vim- Rv* | 5'-ACG AGC CAT TTC CTC CTT CA-3' | | |
| *Slug-Fw* | 5'-GAG TCC ACT GAG TAC CGG AG-3' | | |
| *Slug- Rv* | 5'-CTG CAG ATG AGC CCT CAG A-3' | | |
| *ALDH1A1-Fw* | 5'-TGT TAG CTG ATG CCG ATC TG-3' | | |
| *ALDH1A1-Rv* | 5'-TTC TTA GCC CGC TCA ACA CT-3' | | |
| *CD29-Fw* | 5'-CAT CTG CGA GTG TGG TGT CT-3' | | |
| *CD29-Rv* | 5'-GGG GTA ATT TGT CCC GAC TT-3' | | |
| *EpCAM-Fw* | 5'-CGC AGC TCA GGA AGA ATG TG-3' | | |
| *EpCAM-Rv* | 5'-TGA AGT ACA CTG GCA TTG ACG-3' | | |
| *CD44-Fw* | 5'-AGC AGC GGC TCC TCC AGT GA-3' | | |
| *CD44-Rv* | 5'-CCC ACT GGG GTG GAA TGT GTC T-3' | | |
| *BIP Fw* | 5´-CAT GGT TCT CAC TAA AAT GAA-3´ | | |
| *BIP Rv* | 5´-GCT GGT ACA GTA ACA ACT G-3´ | | |
| *Chop Fw* | 5´-GGA GAA CCA GGA AAC GGA AAC-3´ | | |
| *Chop Rv* | 5´-TCT CCT TCA TGC GCT GCT TT-3´ | | |
