# Supplementary figures and images for: Marigold Supercritical Extract as Potential Co-adjuvant in Pancreatic Cancer: The Energetic Catastrophe Induced via BMP8B Ends Up With Autophagy-Induced Cell Death
Source: Front Bioeng Biotechnol. 2020 Jan 24;7:455. doi: 10.3389/fbioe.2019.00455 (PMC6992545; doi:10.3389/fbioe.2019.00455)

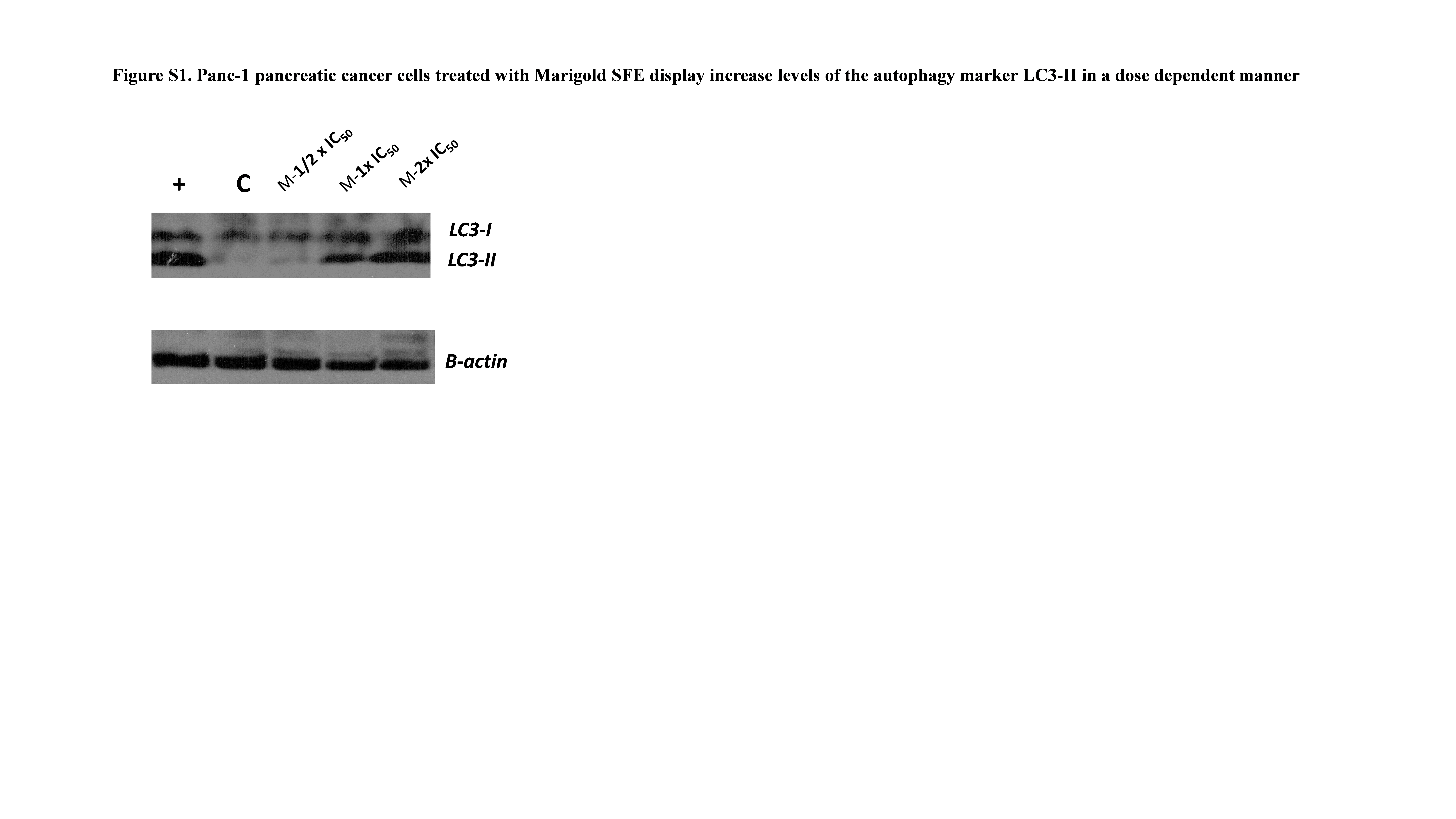

Supplement: Supplementary file 2 [file Image_1.TIF]

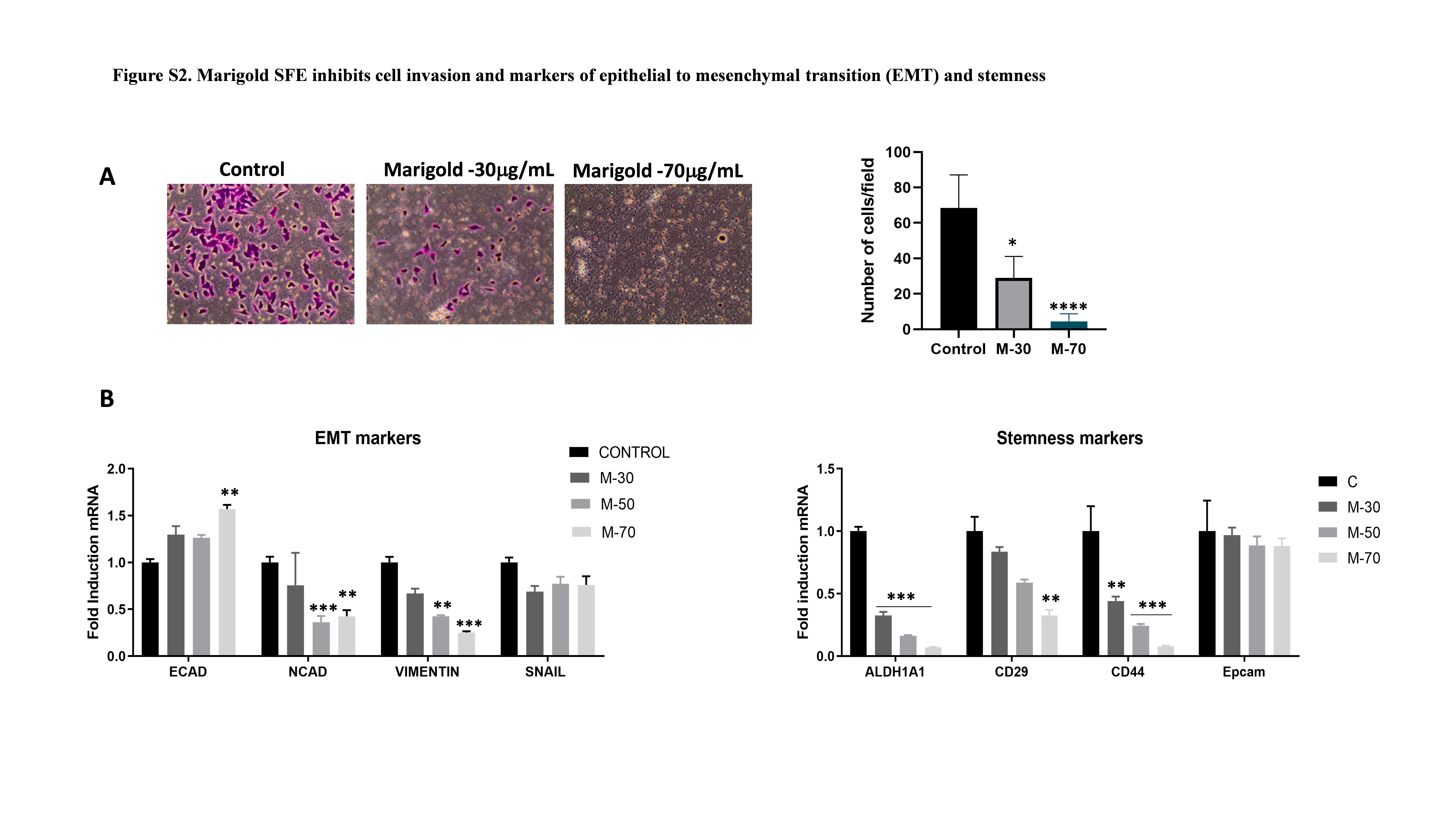

Supplement: Supplementary file 3 [file Image_2.TIF]

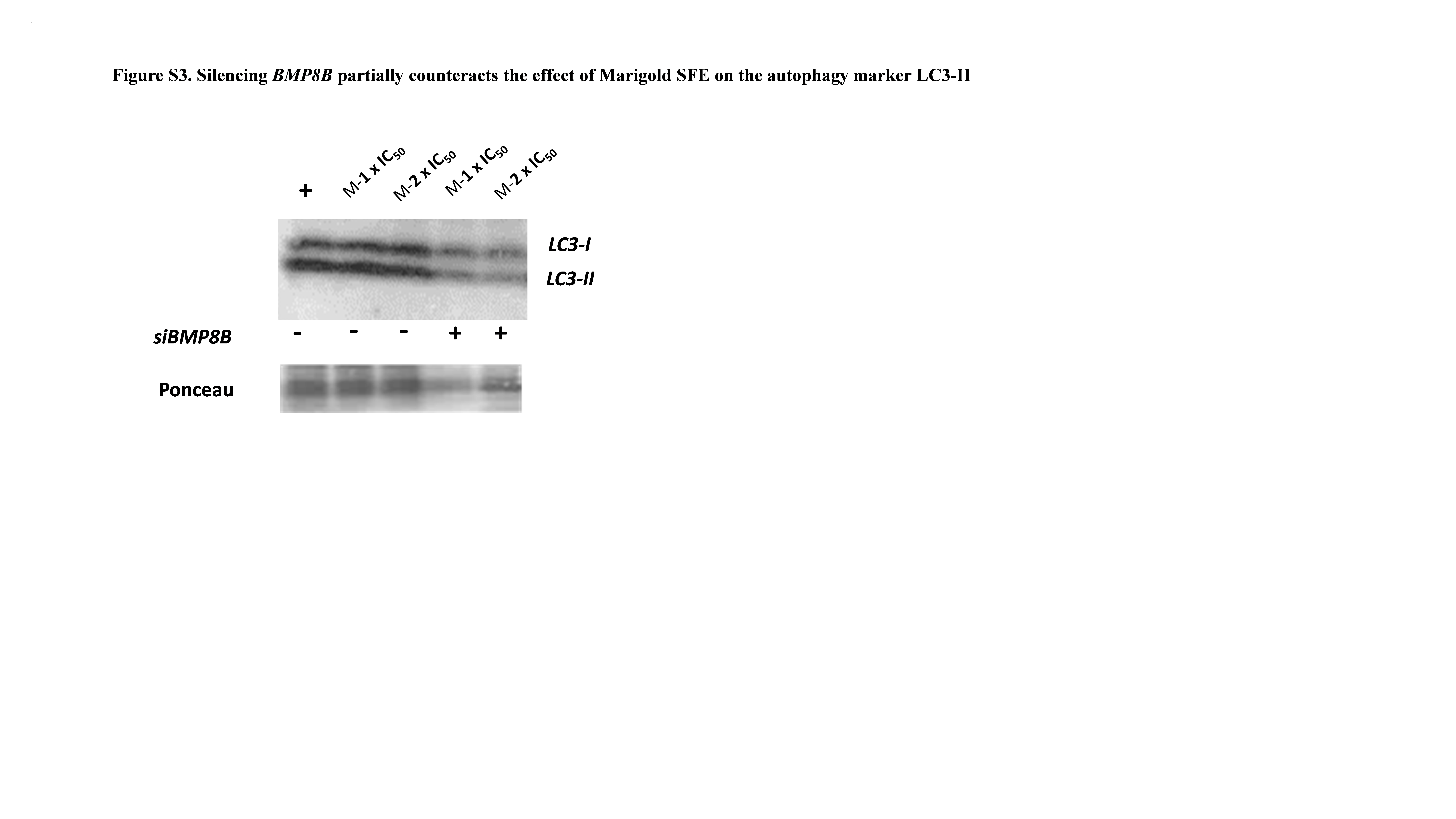

Supplement: Supplementary file 4 [file Image_3.TIF]
